# The Effects of Rho-Associated Kinase (ROCK) Inhibitor Supplementation in Corneal Preservation Media for Donor Tissue Used in Descemet’s Membrane Endothelial Keratoplasty (DMEK)

**DOI:** 10.3390/cells15211944

**Published:** 2026-10-26

**Authors:** Hon Shing Ong, Deepika Malhotra, Dawn J. H. Neo, Xiao Yu Ng, Isha Chaudhari, Gary S. L. Peh, Jodhbir S. Mehta

**Affiliations:** 1 Regenerative Therapy Research Group, Singapore Eye Research Institute, Singapore 169856, Singapore; dawn.neo.j.h@seri.com.sg (D.J.H.N.); ng.xiao.yu@seri.com.sg (X.Y.N.); ishachaudhari1010@gmail.com (I.C.); gary.peh.s.l@seri.com.sg (G.S.L.P.); 2 Singhealth Duke-NUS Ophthalmology & Visual Sciences Academic Clinical Programme, Duke-NUS Graduate Medical School, Singapore 169857, Singapore; 3 Corneal and External Diseases Department, Singapore National Eye Centre, Singapore 168751, Singapore; dpkamalhotra04@gmail.com; 4 Dr. Shroff’s Charity Eye Hospital, Delhi 110002, India

**Keywords:** corneal endothelial cells, endothelial keratoplasty, Descemet’s membrane endothelial keratoplasty (DMEK), Netarsudil, eye banking, Rho-associated kinase inhibitor

## Abstract

Purpose: This study seeks to evaluate the effects of pre-treating donor corneal tissue with a Rho-associated kinase inhibitor (ROCKi), Netarsudil (AR-13324), on the viability of corneal endothelial cells (CECs) during Descemet’s membrane endothelial keratoplasty (DMEK) graft preparation and insertion using endothelium-in and endothelium-out techniques. Methods: This ex vivo comparative study utilized 24 pairs of research-grade human donor corneas preserved in Optisol-GS. One cornea from each pair was incubated in 1 µM AR-13324 for 2 h, while the contralateral cornea served as a control. DMEK tissues were prepared and simulated insertion was performed using either the endothelium-in (Endoglide) or endothelium-out (Geuder glass injector) technique. The surgeon performing the tissue preparation and insertion was masked to the ROCKi treatment allocation. Post-manipulation, tissues underwent live/dead cytotoxicity assay using Calcein-AM and Ethidium homodimer-1 staining. Percentages of live, fallout, and dead CECs were quantified via fluorescence microscopy and ImageJ (version 1.54p) analysis. Correlations between donor age, preservation duration, and cell viability were also analyzed. Results: For the endothelium-out model, live cell percentage was lower in controls compared to ROCKi-treated corneas (89.6 ± 5.35% vs. 95.1 ± 3.74%, *p* = 0.0001), while the percentage of dead cells was higher in controls (7.13 ± 3.71% vs. 2.16 ± 1.39%, *p* = 0.0017). For the endothelium-in model, live cell percentage was lower in controls compared to ROCKi-treated corneas (86.8 ± 4.81% vs. 94.4 ± 3.26%, *p* = 0.0001), with corresponding higher percentage fallout (8.21 ± 5.02% vs. 4.05 ± 2.82%; *p* = 0.0036) and higher percentage dead cells (5.04 ± 3.52% to 1.56 ± 1.13%; *p* = 0.0070). No significant correlations were found between donor age or preservation time and cell viability outcomes. Comparative analysis showed no significant difference in cell survivability between the two DMEK techniques with ROCKi treatment. Conclusions: Pre-incubation with the ROCKi AR-13324 significantly enhances corneal endothelial cell viability and reduces cell death during DMEK graft preparation and manipulation, regardless of the graft insertion technique used. Incorporating ROCKi treatment into tissue preparation protocols may enhance endothelial preservation, potentially expanding its clinical utility in eye banking and improving corneal graft survival.

## 1. Introduction

Corneal clarity and integrity are fundamental to optimal visual function. Corneal endothelial cells (CECs) play a vital role in active fluid regulation to maintain corneal transparency [1]. Human CECs, however, exhibit limited regenerative ability in vivo, leading to irreversible loss of function when damaged due to primary endothelial disorders or secondary endotheliopathies [2]. With the attrition of CECs, as seen in Fuchs endothelial corneal dystrophy (FECD) and pseudophakic bullous keratopathy (PBK), the neighboring cells undergo morphological changes to preserve tissue functionality. This further increases the energy demand on the remaining cells, leading to mitochondrial burnout, oxidative stress, and eventually apoptosis [3,4]. Severe damage to the CECs, ultimately leading to a substantial decrease in cellular densities below a critical threshold, compromises the ability of the remaining CECs to regulate fluid homeostasis in the corneal stroma. This results in corneal decompensation with a loss in its transparency and eventual visual impairment [5]. FECD and PBK are the leading indications for corneal transplantations worldwide.

Over the past 2 decades, endothelial keratoplasties (EKs), namely Descemet’s membrane endothelial keratoplasty (DMEK) and Descemet’s stripping automated endothelial keratoplasty (DSAEK), have progressively overtaken penetrating keratoplasty (PK) as the primary surgical intervention for corneal endothelial disorders due to their faster visual recovery and rehabilitation, reduced surgical astigmatism, and lower rates of rejection [6,7,8]. Without open-sky surgeries, EKs are inherently safer, with lower risks of intraoperative hemorrhage and higher long-term tectonic strength [8]. Between the two EK procedures, DMEK has proven to attain better results in best-corrected visual acuities, faster recovery, and lower rejection rates compared to DSAEK [8,9,10,11]. Multiple studies have also evaluated long-term graft survival and endothelial cell decay following different keratoplasty techniques. Endothelial cell loss (ECL) typically occurs rapidly within the first six months post-transplantation, with losses ranging from 29% to 48%, then transitions to a slower, steadier decrease averaging about 9% per year [12,13,14]. DMEK and DSAEK both show the most significant early ECL, which has been attributed to surgical trauma and inflammation, followed by a slower decline similar to that seen with PK [15,16]. As lower endothelial cell densities (ECDs) at 6 months are linked to higher risks of graft failure across all techniques [14,16,17,18], this highlights the need to minimize perioperative cell loss to enhance long-term graft survival [19].

While DMEK has been reported to achieve good clinical outcomes, it presents technical challenges compared to DSAEK. This includes a steeper learning curve, particularly in DMEK graft preparation, insertion, and intraocular unfolding [8]. There is also a higher incidence of graft detachments, although these risks are dependent on surgical techniques and surgeon experience [20]. Graft manipulation also leads to greater cell loss and can lead to early graft failure [21]. To improve graft survival, current evidence emphasizes the importance of two key measures: rigorous preoperative graft quality assessment and meticulous intraoperative handling of donor tissue to minimize endothelial damage [22]. To reduce the learning curve of surgeons performing DMEK surgeries and to deliver a more predictable and consistent surgical outcome, eye banks have now started preparing pre-stripped and pre-loaded DMEK tissues [23,24]. Utilizing pre-prepared DMEK tissue significantly reduces operative time and minimizes the risk of damaging donor tissue during graft harvesting [13,25].

Amidst the existing global donor shortage, maximizing donor tissue survivability remains an important concern [26]. Rho-associated coiled-coil protein kinases (ROCKs) are serine/threonine kinases that regulate various fundamental cellular processes, such as cell adhesion, motility, proliferation, differentiation, and apoptosis [27,28]. Their roles vary, depending on the type of cells. The latest evidence supports that ROCK inhibition stimulates in vivo CEC proliferation, as well as cellular migration and some form of apoptosis suppression [29,30,31,32]. We have also previously demonstrated the stimulatory effects of ROCK inhibitors (ROCKis) in the pro-proliferative and pro-migratory pathway, showing significant improvements in the attachment and proliferation of CECs and enhanced CEC migration upon adding ROCKi, Y-27632 [29,33]. In addition to the use of Y-27632, studies have been conducted to examine the impact of other ROCKis on corneal endothelial cells [34,35,36,37,38,39]. Our group showed that Netarsudil (AR-13324) exhibited comparable, if not superior, effects on primary human CECs when compared to Y-27632 [34].

At present, ROCKis are approved for the treatment of glaucoma and are being investigated for their potential role in corneal endothelial diseases [34,40]. We hypothesize that ROCKis can potentially enhance corneal endothelial preservation during DMEK. In this study, we investigated the effects of pre-treating donor corneal tissue with the ROCKi Netarsudil (AR-13324) prior to simulating two distinct DMEK tissue loading and insertion techniques: an “endothelium-out” and an “endothelium-in” method [41].

## 2. Methods

### 2.1. Study Design

This was an experimental ex vivo study. The study was designed to determine if pre-treating donor DMEK tissues with ROCKis before preparation and insertion reduced cell dropout and enhanced cell viability. Correlations between donor age and the duration of tissue storage in preservation media (Optisol-GS) with the post-insertion viability of CECs were also analyzed.

### 2.2. Materials

Dulbecco’s phosphate-buffered saline (DPBS), distilled water, OptiMEM-I Reduced Serum Medium (OptiMEM-I), and a LIVE/DEAD™ Viability/Cytotoxicity kit consisting of Calcein AM and Ethidium homodimer-1 (EthD-1) were purchased from ThermoFisher Scientific (Waltham, MA, USA). Trypan blue (0.4%) was purchased from Sigma (St. Louis, MO, USA). Optisol-GS was from Bausch and Lomb (Rochester, NY, USA), and an Endosol balanced salt solution (BSS), an ophthalmic irrigation solution, was from Johnson & Johnson Vision (Irvine, CA, USA). Netarsudil, also known as AR-13324, was obtained from Aerie Pharmaceuticals Inc. (Bedminster, NC, USA). An AR-13324 stock solution (10 mM) was prepared by dissolving the powder in distilled water.

#### 2.2.1. Research-Grade Human Corneoscleral Tissues

A total of 24 pairs of research-grade human cadaver donors were procured for this study through the Lions Eye Institute for Transplant and Research (Tampa, FL, USA), each with a written consent from the next of kin and adhering to the principles outlined in the Declaration of Helsinki. Details of donors are listed in Table 1. Corneoscleral tissues were preserved in Optisol-GS at 4 °C and processed within 21 days of preservation. A ROCK inhibitor was added to one cornea, and the contralateral cornea was used as control. The ROCK inhibitor used in the study was Netarsudil, AR-13324. The preparation of working solutions of Netarsudil, the addition of Netarsudil to Optisol-GS, and tissue handling were all performed under sterile conditions within a biosafety cabinet.

#### 2.2.2. Ex-Vivo Assay

##### Post-Strip DM–Endothelium Incubation in ROCK Inhibitor

For this part of the study, a total of 4 pairs of donor corneas were used. The donor DM–endothelium tissue was first carefully stripped using a standard SCUBA technique [42,43], placed back into the viewing chamber with 15 mL filtered Optisol-GS (topped with 4 mL of fresh Optisol-GS, see below), and supplemented with 1 μM of AR-13324. For the donor-matched contralateral cornea, the donor DM–endothelium tissue was peeled, before being placed back into the viewing chamber containing 19 mL Optisol-GS only, omitting AR-13324 (control). Vehicle controls (water only, in Optisol-GS) were not included within this study due to the high dilution factor utilized (1 in 10,000× dilution), which is unlikely to result in any measurable change to pH and osmolality of Optisol-GS. Donor corneas with their stripped DM–endothelium tissue were kept at 4 °C for 48 h before being pulled through the injector using the endothelium-in model and undergoing a live/dead cytotoxicity assay.

##### Pre-Strip Corneoscleral Tissue Incubation in ROCK Inhibitor

For this segment of the study, corneoscleral tissues were temporarily removed from the viewing chamber and placed in a well of a 12-well plate, endothelium up. Next, 0.5 mL of Optisol-GS was removed from the respective viewing chamber and immediately added onto the endothelium to keep the tissues hydrated. The remaining Optisol-GS from the viewing chamber was filtered through a 0.22 μm syringe filter attached to a 20 mL syringe into a 50 mL falcon tube. The viewing chamber was washed twice with DPBS^-^ and twice with distilled water. Subsequently, 15 mL of the filtered Optisol-GS was added back into the respective chamber. Each sample was then topped up with 4 mL of fresh Optisol-GS. The corneoscleral tissues were placed back in their respective chambers. It should be noted here that one of the corneoscleral tissue samples was supplemented with 1 μM of AR-13324, while the donor-matched contralateral cornea was kept as a control. Vehicle controls (water only, in Optisol-GS) were not included. The cornea pairs were then kept at 4 °C for two hours. This duration of ROCKi supplementation is to keep the protocol feasible in clinical settings.

##### Descemet’s Membrane (DM) Stripping

After two hours, DM stripping of the corneas was performed using the same SCUBA technique [42,43]. Briefly, the corneoscleral tissue was placed on a vacuum holder (CORONET^®^, Network Medical, Ripon, UK) and the stripping was performed under a stereomicroscope (Nikon SMZ1270, Tokyo, Japan). The DM–endothelium was stained with 0.2% Trypan Blue (1:1 dilution from a 0.4% stock, T8154, Sigma-Aldrich, Burlington, MA, USA) and the dye was replaced with Optisol-GS after brief staining. Careful separation of the DM–endothelium was performed with a sinskey hook. After ensuring that the DM had separated from the stroma bed, the DM was peeled gently using fine non-toothed forceps from one end.

##### Endothelium-Out Model

The DM–endothelium scroll, with the endothelium on its outer surface, was placed into a balanced salt solution (BSS). The glass injector system (Geuder AG, Heidelberg, Germany) was created using the glass cannula attached to a tube with a syringe on one side. The DM–endothelium scroll was aspirated from the broad opening of the glass injector cannula out of the BSS. After aspiration, the opening was closed with a 3 mL syringe fully filled with BSS. Once loaded, the DM–endothelium scroll was injected onto a glass slide from the narrow end of the glass injector cannula. A total of N = 10 matched pairs of corneas were used for this model.

##### Endothelium-In Model

The donor DM–endothelium tissue was folded into a trifold, with the endothelium on the inner side. After the trifold, the tissue was loaded into the DMEK EndoGlide™ (CORONET^®^, Network Medical, Ripon, UK). Using a 23 G forceps, the DM–endothelium tissue was pulled through the injector and released directly on a glass slide [44]. A total of N = 10 matched pairs of corneas were used for this model.

All procedures, DM stripping, loading, and pull-through were performed by a single experienced surgeon, HSO. The surgeon was masked to the experimental groups.

#### 2.2.3. Live/Dead Cytotoxicity Assay

Stock solutions of Calcein AM (4 mM) and Ethidium homodimer-1 (2 mM) were diluted in 1x phosphate buffered saline (PBS) to give a combined working solution containing 4 μM of each reagent. Then, 100 μL of the working solution was immediately applied to the DM–endothelium tissue. The slide holding the DM–endothelium was then placed in a 4-well rectangle dish and incubated at 37 °C with 5% CO_2_ for 30 min. The live/dead stain was replaced with 1x PBS before imaging.

#### 2.2.4. Microscopy Imaging

Images of the stained DM–endothelium were taken on a Zeiss Axio Imager.M2m (Oberkochen, Germany). Five images at 10x magnification were acquired centrally, taking care to avoid the peripheral edges to avoid discrepancies due to edge handling and tissue manipulation from slide mounting and imaging. For each of the images, five regions of interest (ROI) of 250 × 250 μm were analyzed at standardized predefined locations, avoiding the edges: top left, center, top right, bottom left, and bottom right of image. The images of each area were stacked using the ImageJ (version 1.54p) plug-in, Stackfocuser [45]. Cell count was then performed using the ImageJ multi point tool [45]. Green fluorescent cells positive for Calcein-AM were counted as the live cells, red fluorescent cells positive for EthD-1 were counted as dead cells and black areas were counted for cell dropout. The counts were tabulated to obtain the percentage of live, dead and fallout cells.

#### 2.2.5. Statistical Analysis

Statistical analysis was performed in Graphpad Prism 9.0 unless otherwise stated. As two corneas from each donor were used (paired donor design), paired Student’s *t*-tests were performed to ensure there were no baseline differences in the endothelial cell density of the right and left eyes. The proportions of live, fallout, and dead cells (primary outcome) were quantified and expressed as a percentage of total cells (mean ± SD). A Pearson’s correlation test was also performed to assess the strength and direction of the linear relationship between donor age, sex, and preservation-to-processing time with the percentages of live, fallout, and dead cells. *p*-values of <0.05 were considered as statistically significant. A multivariable analysis was performed to evaluate the correlation between donor age, sex and preservation to processing time with the percentage of live, fallout, and dead cells.

## 3. Results

### 3.1. Donor Information

**Figure 1:**
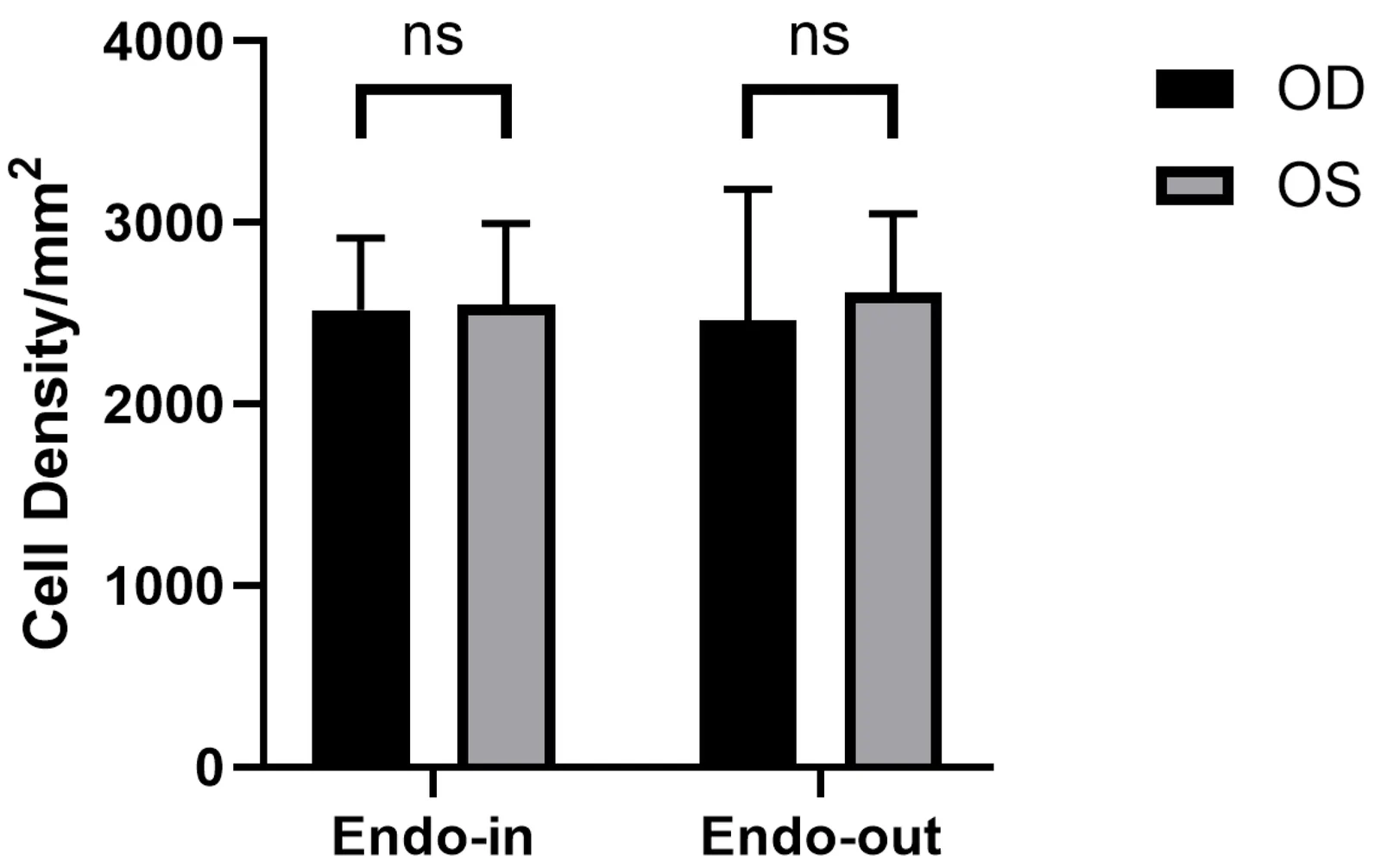
Baseline comparison of paired donor tissue (OD versus OS) showed no significance between OD and OS endothelial cell density for both endothelium-in (endo-in) and endothelium-out (endo-out) experiments conducted. Paired Student’s *t*-test was used. ns: not statistically significant.

The study utilized 24 pairs of research-grade human corneoscleral tissue, with donors ranging in age from 43 to 67 years and endothelial cell densities (ECDs) varying between 1232 and 3597 cells/mm^2^. The tissues were preserved in Optisol-GS for durations ranging from 8 to 21 days before processing. Table 1 summarizes the donor characteristics, showing balanced demographics across the two techniques: endothelium-in (DMEK Endoglide) and endothelium-out (Geuder glass injector). There were no significant differences between the baseline endothelial cell density of the OD and OS tissue from each donor (Figure 1).

**Figure 2:**
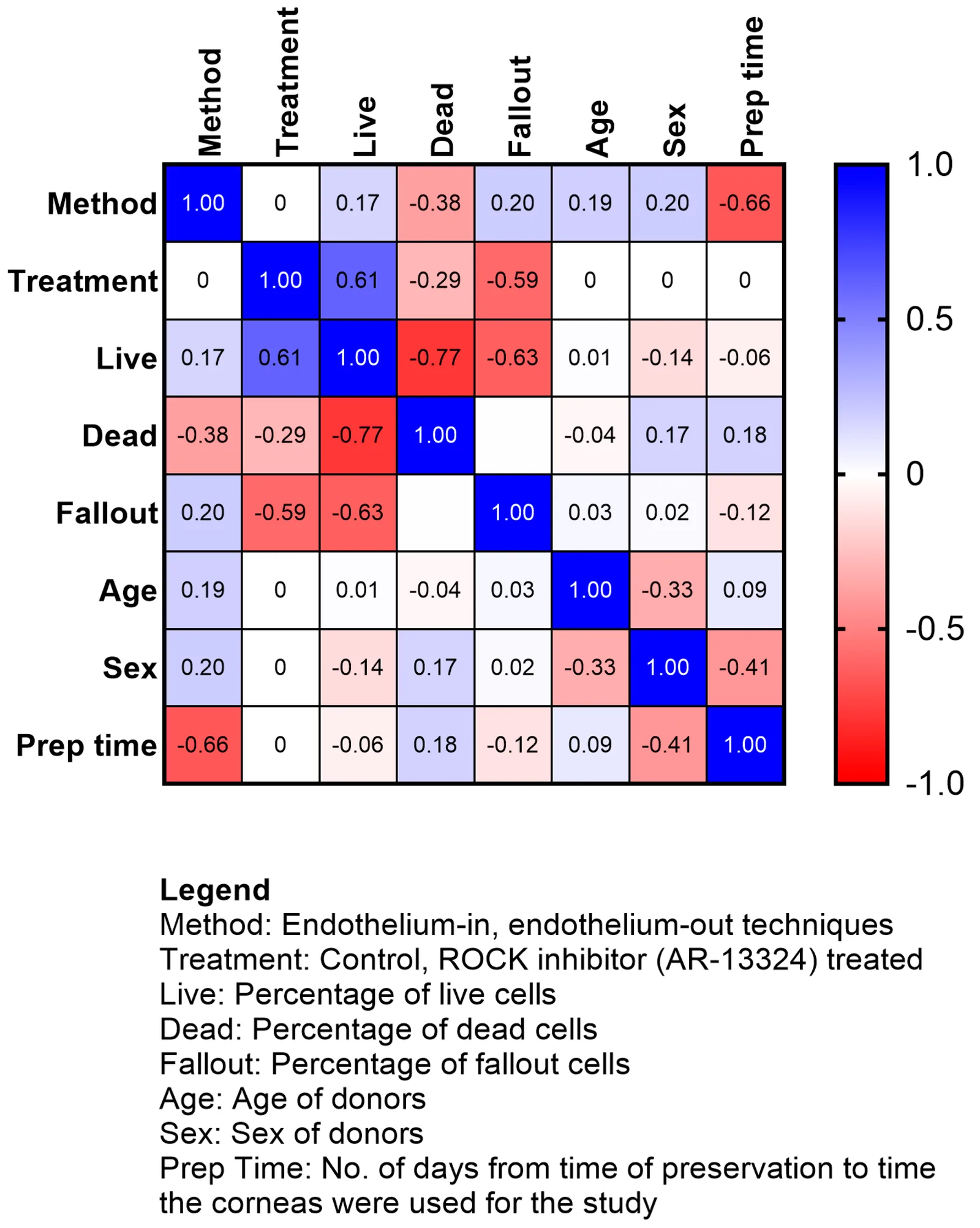
Multivariable analysis of parameters in the study that could have influenced the outcome. This figure presents a correlation matrix heatmap, which displays Pearson’s correlation coefficients (r) calculated between all variables, using data from both endothelium-in and endothelium-out techniques. Values between |0.5| and |1.0| suggest a strong correlation between factors; values between |0.3| and |0.49| indicate a moderate correlation, and values below |0.29| indicate a weak correlation between variables; 0 implies no relationship between variables.

A multivariable analysis was also performed to evaluate any correlation between donor age, sex and preservation to processing time with the percentage of live, fallout and dead cells (Figure 2). The weak correlation between the variables suggests that there was little to no relation between these parameters. There was no statistically significant correlation between age and outcomes (live, fallout, dead) in either the control or treated group. In the control group, age showed a moderate negative correlation with percentage of live cells (r = –0.522, *p* = 0.121) and moderate positive correlations with percentage of fallout (r = 0.409, *p* = 0.241) and percentage dead cells (r = 0.345, *p* = 0.329). Similarly, in the treated group, age demonstrated a weak negative correlation with percentage of live cells (r = –0.328, *p* = 0.355) and a weak positive correlation with percentage of fallout (r = 0.397, *p* = 0.257) and percentage of dead cells (r = 0.0218, *p* = 0.952).

There was no statistically significant correlation between preservation time and outcome measures (live, fallout, or dead) in either the control or treated groups (all *p* > 0.05). Although a weak positive trend was noted between preservation time and live outcomes in the treated group, the correlation was not statistically significant, suggesting that preservation time had no consistent impact on outcome measures.

### 3.2. Initial 48-h Incubation in ROCKi

**Figure 3:**
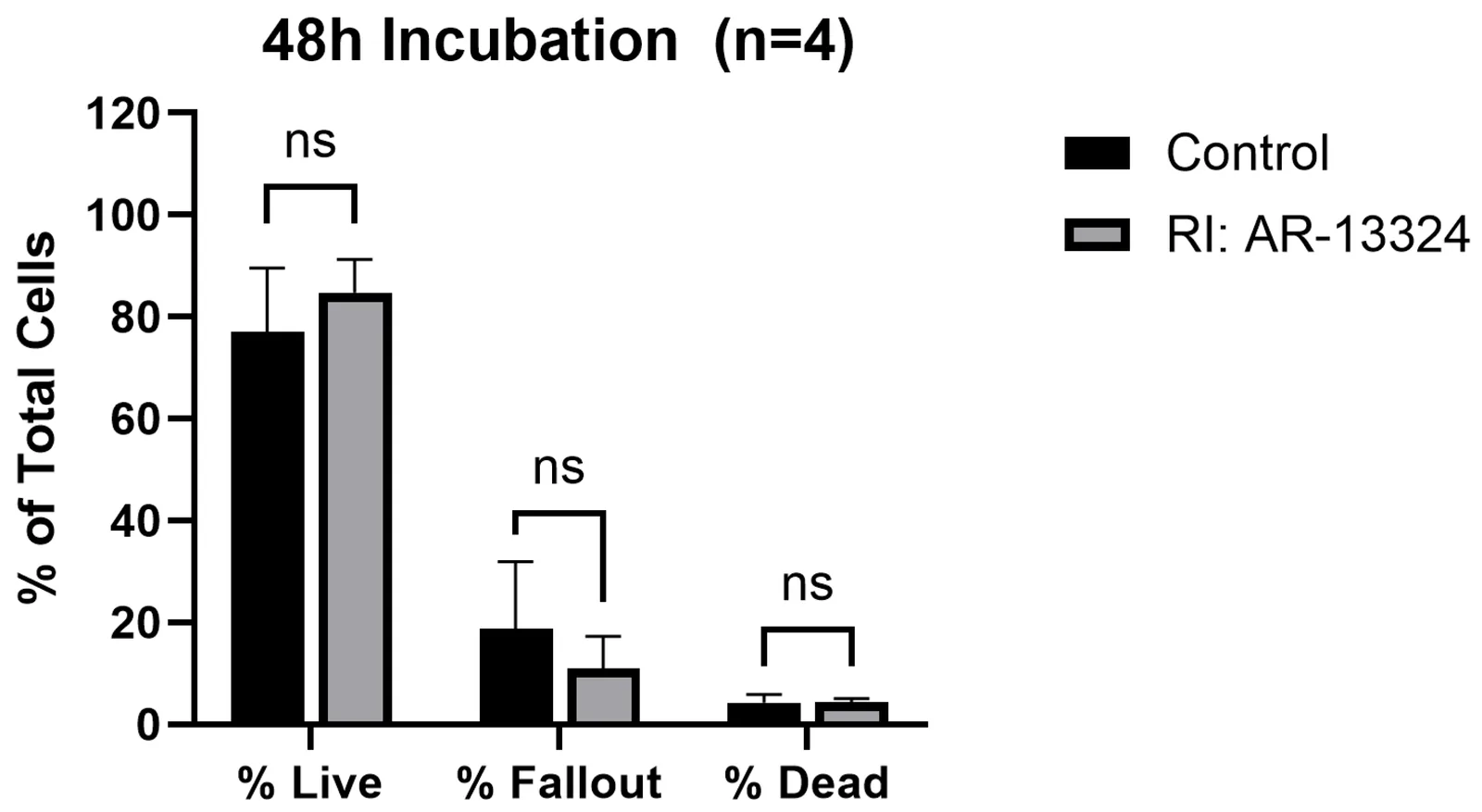
AR-13324 incubated tissue versus controls in a 48 h incubation optimization model. Results showed that AR-13324 incubation of corneas up to 48 h was non-detrimental to cells. No significant difference between controls and ROCKi-incubated (RI) tissues were observed. A total of n = 4 pairs were used. ns: not statistically significant.

Treated tissues were incubated with 1 μM of AR-13324 based on results reported in our earlier publication [29]. Through the 48 h incubation, it was determined that the duration of ROCKi incubation of up to 48 h was non-detrimental to the cells (Figure 3). Following an endothelium-in pull-through technique, there were no significant differences in the percentage of live, dead, and fallout cells in the tissues that were incubated in the ROCKi when compared to controls. To keep the protocol feasible in clinical settings, we reduced the duration of incubation to 2 h for the rest of the experiment to evaluate the efficacy of ROCKi incubation prior to DMEK graft stripping, loading, and pull-through.

### 3.3. Effects of 2-h Incubation in ROCKi on DMEK Tissues

#### 3.3.1. Endothelium-Out Technique (Geuder Glass Injector)

##### Donor Characteristics

The mean age of donors was 58.9 ± 7.8 years, with a range of 45 to 67 years. Among these, 6 of the 10 matched pairs were from female patients. Time from preservation to processing ranged between 8 and 14 days.

##### Viability Analysis

**Figure 4:**
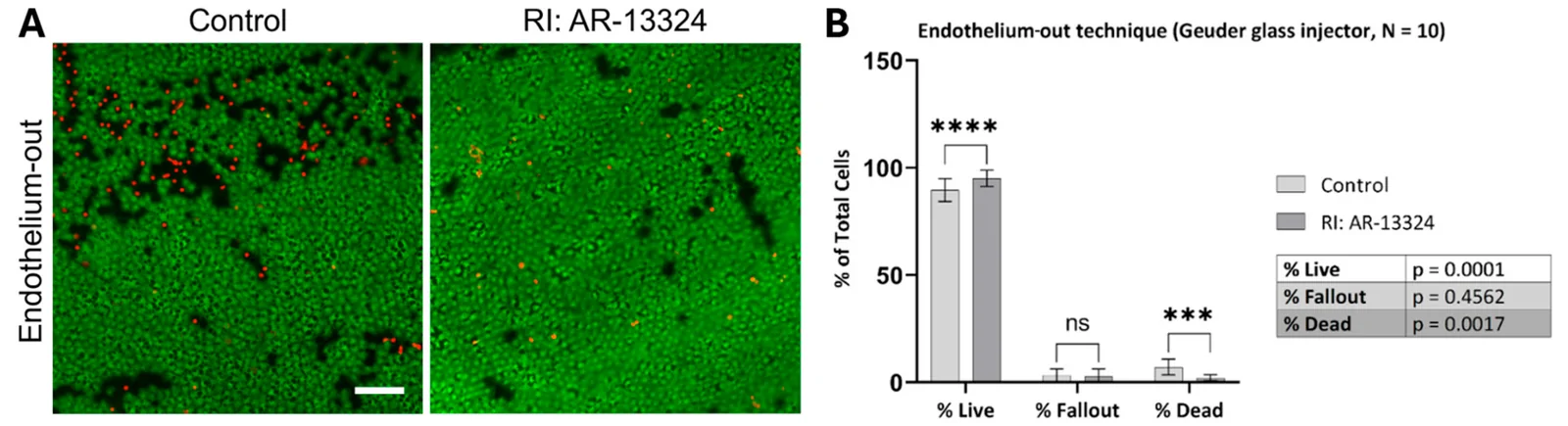
Ex vivo corneal endothelial cell viability assay post-Descemet’s stripping with the endothelium-out technique showed greater survivability and decreased death of corneal endothelial cells (CECs) with AR-13324 incubation. N = 10 matched pair donor corneas were treated with 1 μM AR-13324 prior to DM stripping using the endothelium-out technique with the Geuder glass injector. Fluorescent green cells represent live cells, fluorescent red cells represent dead cells, and black areas represent cell dropout. (**A**) Representative images showing live/dead cell viability stains on tissue with and without pre-incubation with AR-13324 and following Descemet’s stripping endothelium-out (Geuder glass injector) technique. Scale bar represents 100 μm. (**B**) Percentages of live, fallout, and dead cells in the control group were 89.60 ± 5.35%, 3.27 ± 3.02%, and 7.13 ± 3.71%, respectively. Percentages of live, fallout, and dead cells in the treated group were 95.14 ± 3.74%, 2.70 ± 3.52%, and 2.16 ± 1.39%, respectively. Data are shown as mean ± SD, ****/*** *p* < 0.01/0.001. RI: ROCK inhibitor; ns: not statistically significant.

Corneal endothelial cell viability was assessed after utilizing the endothelium-out technique with the Geuder glass injector to release the tissue onto a glass slide. Cell viability was assessed by live/dead staining and quantified as the percentage of live, fallout, and dead cells (Figure 4). The treated group (with AR-13324) showed a significantly higher percentage of live cells (95.1 ± 3.74%) compared to the control group (89.6 ± 5.35%), *p* = 0.0001. The proportion of dead cells was markedly lower in the treated corneas (2.16 ± 1.39%) compared to controls (7.13 ± 3.71%), *p* = 0.0017. The percentage of fallout was comparable between groups, 3.27 ± 3.02% in the control group and 2.70 ± 3.52% in the treatment group, with no statistically significant difference.

To determine whether donor age or preservation time influenced cell viability, correlation analyses were performed separately for control and treated samples (Supplementary Figure S1). Across all age groups, the percentages of live, fallout, and dead cells showed no statistically significant correlations with age in either control (live: *r* = −0.522, *p* = 0.1215; fallout: *r* = 0.409, *p* = 0.241; dead: *r* = 0.345, *p* = 0.3289) or treated samples (live: *r* = −0.328, *p* = 0.3554; fallout: *r* = 0.397, *p* = 0.257; dead: *r* = 0.0218, *p* = 0.952). Similarly, no significant correlations were observed between days from preservation and cell viability parameters for either group (control—live: *r* = −0.0609, *p* = 0.867; fallout: *r* = 0.455, *p* = 0.187; dead: *r* =−0.375, *p* = 0.285; treated—live: *r* = 0.680, *p* = 0.0979; fallout: *r* = −0.0667, *p* = 0.8548; dead: *r* = 0.0621, *p* = 0.865). Overall, neither age nor preservation duration significantly affected the proportions of live, fallout, or dead cells, and treatment did not alter these relationships.

#### 3.3.2. Endothelium-In Technique (DMEK Endoglide)

##### Donor Characteristics

The mean donor age was 56.0 ± 8.3 years, with a range of 43 to 67 years. Among these, four of 10 matched pairs were from female patients. Time from preservation to processing ranged between 10 and 21 days.

##### Viability Analysis

**Figure 5:**
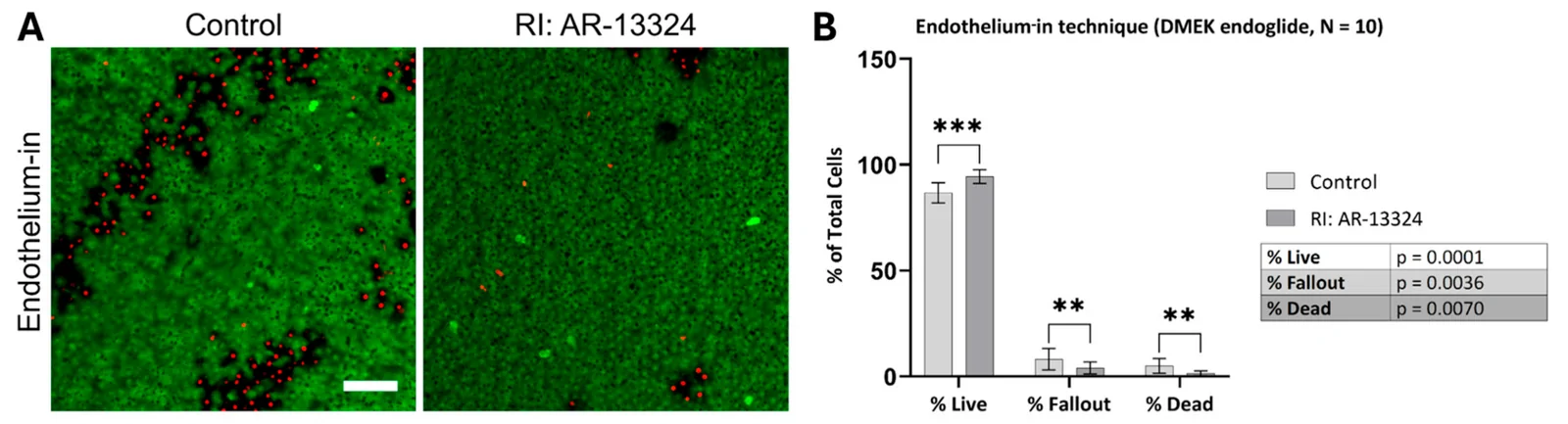
Ex vivo corneal endothelial cell viability assay post-Descemet’s stripping using the endothelium-in technique showed greater survivability, reduced fallout, and decreased death of CECs with AR-13324 incubation. N = 10 matched pair donor corneas were treated with 1 μM AR-13324 prior to DM stripping using the endothelium-in technique with the DMEK endoglide. Fluorescent green cells represent live cells, fluorescent red cells represent dead cells, and black areas represent cell dropout. (**A**) Representative images showing live/dead cell viability stains on tissue with and without pre-incubation with AR-13324 and following Descemet’s stripping endothelium-in (Endoglide) technique. Scale bar represents 100 μm. (**B**) Percentages of live, fallout, and dead cells in the control group were 89.60 ± 5.35%, 3.27 ± 3.02%, and 7.13 ± 3.71%, respectively. Percentages of live, fallout, and dead cells in the control group were 86.75 ± 4.81%, 8.21 ± 5.02%, and 5.04 ± 3.52%, respectively. Percentages of live, fallout, and dead cells in the treated group were 94.39 ± 3.26%, 4.05 ± 2.82%, and 1.56 ± 1.13% respectively. Data are shown as mean ± SD, **/*** *p* < 0.01/0.001, respectively. RI: ROCK inhibitor.

Corneal endothelial cell viability was assessed after utilizing the DMEK Endoglide technique to release the tissue onto a glass slide. Cell viability was assessed by live/dead staining and quantified as the percentage of live, fallout, and dead cells. As shown in Figure 5, AR-13324 treatment resulted in a significantly higher percentage of live cells (control: 86.8 ± 4.81%; treated: 94.4 ± 3.26%; *p* = 0.0001) and a reduced percentage of fallout (8.21 ± 5.02% vs. 4.05 ± 2.82%; *p* = 0.0036) and dead cells (5.04 ± 3.52% vs. 1.56 ± 1.13%; *p* = 0.0070).

Correlation analyses revealed no significant linear relationships between either age or preservation time and outcome measures (live, fallout, or dead) in either the control or treated groups (Supplementary Figure S2). In the control group, correlations between age and outcomes ranged from *r* = –0.29 to +0.42 (*p* = 0.22–0.57), while in the treated group they ranged from *r* = –0.38 to +0.39 (*p* = 0.27–0.40). Similarly, preservation time showed weak correlations with outcome measures in both groups (control: *r* = –0.39 to +0.28, *p* = 0.26–0.57; treated: *r* = –0.08 to +0.05, *p* = 0.83–0.90). These results indicate that neither age nor preservation time was significantly associated with live, fallout, or dead outcomes under either experimental condition.

A comparison between the control and ROCKi-treated groups for the two different techniques showed no significant difference between the endothelium-in and endothelium-out techniques (Supplementary Figure S3).

## 4. Discussion

This study evaluated the effects of short-term pre-incubation of donor corneal tissues with the ROCKi Netarsudil (AR-13324) on endothelial cell survival during DMEK tissue preparation. Two widely used graft loading and insertion techniques, endothelium-in (EndoGlide) and endothelium-out (Geuder glass injector) models, were simulated. Our findings demonstrated a clear cytoprotective effect of ROCKi supplementation in both models. Compared to paired control corneas, tissues that were incubated in the ROCK inhibitor for two hours showed a significantly higher proportion of viable cells and fewer dead or fallout cells following manipulation. In the endothelium-out model, live cell percentages increased from 89.6 ± 5.3% to 95.1 ± 3.7% (*p* = 0.0001) and dead cells reduced from 7.1 ± 3.7% to 2.2 ± 1.4% (*p* = 0.0017). Similarly, in the endothelium-in model, live cell survival improved from 86.8 ± 4.8% to 94.4 ± 3.3% (*p* = 0.0001), while fallout and dead cells were significantly reduced (*p* = 0.0036 and *p* = 0.0070, respectively). Importantly, donor age and preservation duration showed no significant correlation with post-insertion viability in the either control or treated groups, suggesting that the beneficial effect of ROCK inhibition was consistent across donor variables. Collectively, these data indicate that a minimum of 2 h of short-term exposure to 1 µM AR-13324 provides measurable protection against manipulation-induced endothelial injury in donor DMEK tissues. In the post-strip DM–endothelium incubation study, it was evident that a 48-h incubation did not result in detrimental effects on CECs.

The underlying mechanism for this protective effect likely involves the role of ROCK in mediating cytoskeletal organization and amplifying stress-induced apoptosis [24,25,29,37,38]. By inhibiting ROCK, AR-13324 may help preserve endothelial cell structure and attenuate apoptotic signaling during tissue manipulation and insertion, which represent periods of substantial cellular stress for donor endothelial cells. ROCKis have been extensively investigated for their ability to enhance the adhesion of CECs, their migration, and cellular proliferation [29,32]. Okumura et al. demonstrated that injecting cultivated CECs supplemented with the ROCKi Y-27632 improved cell adhesion in animal models of endothelial dysfunction, with increased ZO-1 and Na^+^/K^+^-ATPase expression indicating functional recovery [46]. In the same series of studies, topical or intracameral administration of Y-27632 was shown to accelerate corneal endothelial wound healing, resulting in significantly higher endothelial cell densities, improved corneal clarity, and reduced corneal thickness, likely due to enhanced cellular migration and pump function [47,48]. Similarly, we have previously showed that the pre-treatment of cultured primary CECs with Y-27632 conferred a pro-survival trend when these CECs were dissociated for expansion [29]. These findings collectively established ROCK inhibition as a promising strategy for both cell-based therapy and tissue regeneration in corneal endothelial disorders.

More recently, our group compared multiple ROCKis—including Netarsudil (AR-13324)—in cultured primary human CECs [34]. This study revealed that AR-13324 promoted cell proliferation and migration at low concentrations, showing effects comparable to, or exceeding, Y-27632. However, higher doses or prolonged exposure led to dose-dependent cytotoxicity, emphasizing the need to balance potency with safety. In contrast, our study demonstrated that a minimum of 2 h of short-term incubation of intact donor tissue with 1 µM AR-13324 was non-toxic and beneficial, suggesting that brief ex vivo exposure can safely leverage its cytoprotective properties.

Clinical evidence further supports the translational potential of ROCKi in promoting endothelial recovery. In patients with FECD, ripasudil 0.4% used as an adjuvant to Descemet Stripping Only (DSO) improved endothelial cell recovery, enhanced visual acuity, and increased corneal clarity at one year post-treatment [49,50]. Price et al. similarly reported that Netarsudil 0.02% eyedrops significantly reduced central corneal thickness and improved best-corrected visual acuity in symptomatic FECD patients after three months [38].

These findings, together with our ex vivo results, strengthen the evidence that ROCKis enhance endothelial cell function and integrity through mechanisms that promote adhesion, cytoskeletal stability, and survival. While clinical trials have primarily used topical or intracameral formulations, our data suggest that ex vivo donor tissue preconditioning with ROCK inhibitors could serve as a complementary strategy to further improve outcomes in endothelial keratoplasty. This is the first known report demonstrating that a brief 2-h exposure to a ROCKi improves endothelial viability prior to mechanical manipulation in DMEK preparation, an important step that could potentially enhance graft survival.

Through a systematic review, endothelial cell loss was significantly lower in “Endothelium-in” DMEK surgeries at six months post-operation compared to “Endothelium-out” surgeries, with comparable visual outcomes and complication rates [41]. Nevertheless, the endothelium-in approach requires additional tissue manipulation during trifold preparation, which has been suggested as a potential source of endothelial injury [51,52]. Notably, focal patterns of endothelial cell loss associated with forceps manipulation and trifold formation have been described [52]. However, previous ex vivo studies have generally demonstrated comparable or even lower overall endothelial cell loss compared with endothelium-out techniques [51,52,53], with Chong et al. reporting greater overall endothelial viability with the endothelium-in approach h [54].

In the present study, ROCKi treatment appeared to further mitigate endothelial injury associated with tissue manipulation and insertion. In the untreated control group, cellular fallout was higher with the endothelium-in technique (*p* = 0.015), whereas no significant difference was observed between techniques following ROCKi treatment. These findings suggest that ROCKi may enhance endothelial resilience to mechanical stress during graft preparation and insertion, thereby reducing differences in endothelial cell loss between the two techniques. While the endothelium-in approach has demonstrated potential advantages in terms of graft handling and unfolding [8,41], endothelium-out remains the more widely adopted technique because of its familiarity and established clinical outcomes. Taken together, our findings suggest that ROCKis may provide endothelial protection during DMEK regardless of the preparation and insertion technique used.

The demonstrated cytoprotective effect of AR-13324 on donor endothelial tissue has great practical implications for eye banking and corneal transplantation. DMEK surgery outcomes are heavily dependent on the health and density of the donor endothelium [21]. Pre-incubation with a ROCKi could help reduce cell loss during graft preparation, shipment, or intraoperative manipulation—particularly for pre-stripped or pre-loaded DMEK tissues. Integration of a brief ROCKi incubation step could be readily adopted without significantly impacting surgical logistics. These benefits could contribute to alleviating the global shortage of donor corneal tissue by maximizing the survival of available grafts [26]. By reducing cell loss during graft preparation and insertion, this approach may improve graft survival rates and reduce the risk of early failure, potentially extending the viable storage time for donor tissues and resulting in better quality grafts, especially in the context of pre-stripped and pre-loaded tissue. This may also benefit surgeons new to DMEK surgeries who encounter more difficulties during graft preparation and manipulation. Although we showed that a 48-h incubation of the ROCKi did not have detrimental effects on CECs, no significant improvement in the overall cell counts of the tissues in the presence of the ROCKi was observed for either insertion approach. This is an interesting observation considering that both preparation processes involved mechanical manipulation of the graft material that can stress and damage the corneal endothelium. Perhaps this was because all tissue preparation and handling procedures were performed by a single experienced surgeon, which may have minimized endothelial damage, hence attenuating the observable protective effect of ROCK inhibition. In less experienced hands, under suboptimal conditions, or in unexpected situations, trauma to the graft may lead to increased cell damage, wherein the protective effect of a ROCKi may be more noticeable, potentially preventing substantial endothelial cell loss. Taken together, a 48-h incubation with a ROCKi potentially supports the feasibility of overseas tissue transport in media supplemented with a ROCK inhibitor, where extended preservation is required. This could be particularly advantageous for pre-loaded DMEK tissue, where the protective effects of ROCK inhibitors on endothelial cells could mitigate the negative impact of prolonged storage times during the transport of pre-loaded DMEK tissues.

In a broader context, our findings complement existing clinical data on topical ROCKi in FECD and endothelial dysfunction, suggesting that pharmacologic modulation of endothelial behavior, whether in vivo or ex vivo, may play a central role in the next generation of corneal endothelial therapies. Future work should focus on correlating ex vivo viability with in vivo graft performance, evaluating postoperative endothelial density, and exploring temperature-controlled, standardized ROCKi donor pre-treatment protocols suitable for routine eye banking and clinical use.

While promising, this study has several limitations. First, the experiments were performed on ex vivo donor tissue, which provides a controlled setting but cannot replicate the full physiological and metabolic environment of the anterior chamber. Second, although the paired-eye design minimized donor-related variability, larger datasets are needed to confirm subtle donor-related trends. Third, the main protocol adopted a 2-h exposure to ensure feasibility within clinical eye banking workflows; although a 48-h incubation was initially tested in four pairs without adverse effects, these results do not fully capture longer-term effects, if any. Fourth, tissue handling in open containers, temperature changes during tissue removal, incubation at room temperature, and re-immersion in cold storage could influence endothelial metabolism and viability. Previous studies show that temperature fluctuations can increase endothelial stress and apoptosis, and prolonged room-temperature exposure during tissue processing has been linked to higher risk of fungal contamination [55,56,57], suggesting that open-container time at an ambient temperature is not benign [58]. Fifth, while 1 µM AR-13324 was chosen based on the available literature, no consensus currently exists regarding the optimal ex vivo concentration for corneal applications. Given the potential for concentration-dependent cytotoxicity seen in culture systems, dose–response optimization studies are warranted to define the safest and most effective range for clinical translation. Fifth, the corneas used in this study were research-grade donor tissues and were not intended for clinical transplantation. Accordingly, corneas with ECDs below 2000 cells/mm^2^ were included. Importantly, baseline ECDs did not differ significantly between the treatment and control groups, reducing the likelihood that differences in baseline endothelial cell density confounded the comparison between groups. Nevertheless, we acknowledge that the inclusion of corneas with a wide range of ECDs may limit the direct generalizability of our findings to donor corneas meeting clinical criteria for DMEK. Finally, the long-term effects of AR-13324 on CEC function and graft outcomes were not assessed. Future research should focus on the investigation of potential synergistic effects with other protective agents or storage solutions and incorporate long-term follow-up to assess graft survival and functional outcomes.

## 5. Conclusions

This study demonstrates that short-term pre-treatment of donor corneal tissues with a ROCKi significantly improves endothelial cell survival during simulated DMEK tissue preparation, loading and insertion, regardless of loading configuration. The observed cytoprotective effects of AR-13324 highlights its potential as a clinically applicable adjunct in eye banking and endothelial transplantation protocols, paving the way for future studies correlating ex vivo outcomes with in vivo graft survival and postoperative endothelial function. Further research is warranted to translate these findings into clinical practice and potentially contribute to addressing the global shortage of donor corneal tissue.

## Figures and Tables

**Table 1:** Donor information for corneas procured for the ex vivo assay with Descemet’s membrane stripping, loading, and pull-through using either the endothelium-in (DMEK endoglide) or endothelium-out (Geuder glass injector) technique.

	Endothelium-In Technique (DMEK Endoglide)					Endothelium-Out Technique (Geuder Glass Injector)			
Donor Pairs	Age	Gender	Endothelial Cell Density (ECD) OD/OS	Time from Preservation to Processed (Days)	Donor Pairs	Age	Gender	Endothelial Cell Density (ECD) OD/OS	Time from Preservation to Processed (Days)
1	53	M	2899/3077	14	15	67	F	1383/2088	10
2	60	M	2188/2174	13	16	61	M	2378/2493	14
3	60	F	2101/2101	10	17	64	F	2241/2137	10
4	45	F	2681/2469	18	18	62	M	1232/2392	9
5	60	F	3061/3144	11	19	58	F	2857/2488	9
6	43	M	2467/2992	12	20	45	F	3597/3436	8
7	48	F	2892/2728	17	21	47	F	3125/2801	8
8	65	M	1853/1853	16	22	54	F	2463/2364	8
9	59	M	2733/2685	16	23	65	M	2714/2812	10
10	67	M	2294/2254	21	24	66	M	2639/3155	14
11	70	M	2017/2575	12					
12	62	M	2127/2049	9					
13	60	F	1285/1119	18					
14	68	F	1828/1242	17					

## Data Availability

All data supporting the findings of this study are available within the paper and its Supplementary Information.

## Supplementary Materials

The following supporting information can be downloaded at: https://www.mdpi.com/article/10.3390/cells15211944/s1. Supplementary Figure S1: AR-13324-treated tissues showed higher survivability and less fallout using the Endothelium-out technique (Geuder glass injector) regardless of age or% days in preservation media. (A–C) Scatterplots depict percentage of live, fallout, and dead cells relative to total cell count following the procedure, against donor age. (D–F) Scatterplots depict live, fallout, and dead cell percentage against the number of days in preservation media before the day of procedure. Solid symbols indicate control tissues while outlined symbols represent treated tissues. Pearson correlation coefficients and respective *p*-values are given in the table above. N = 10 matched paired donors were used. The tissues were stained with Calcein-AM and Ethidium homodimer-1 following the procedure and analyzed using ImageJ; Supplementary Figure S2: AR-13324-treated tissues showed higher survivability and reduced fallout using the Endothelium-in technique (DMEK endoglide) regardless of age or days in preservation media. (A–C) Scatterplots depict percentage of live, fallout, and dead cells relative to total cell count following the procedure, against donor age. (D–F) Scatterplots depict live, fallout, and dead cell percentage against the number of days in preservation media before the day of procedure. Solid symbols indicate control tissues while outlined symbols represent treated tissues. Pearson correlation coefficients and respective *p*-values are given in the table above. N=10 matched paired donors were used. The tissues were stained with Calcein-AM and Ethidium Homodimer-1 following the procedure and analyzed using ImageJ; Supplementary Figure S3. Comparison of normalized values between the endothelium-in (Endo-in) and endothelium-out (Endo-out) methods for the AR-13324 treated group; Supplementary Figure S4. Example of live/dead cell viability staining for corneal endothelial cell viability post-Descemet’s stripping using Calcein-AM and Ethidium homodimer-1. (A) Stitched whole image of stained graft. (B–D) Inlays showing respective regions of interest with dots indicating (B) live cells (green fluorescence), (C) dead cells (red fluorescence), and (D) fallout (empty areas). Scale bar represents 100 μm.
