# Supplementary material for: The Effects of Rho-Associated Kinase (ROCK) Inhibitor Supplementation in Corneal Preservation Media for Donor Tissue Used in Descemet’s Membrane Endothelial Keratoplasty (DMEK)
Source: Cells. 2026 Oct 26;15(21):1944. doi: 10.3390/cells15211944 (PMC13762406; doi:10.3390/cells15211944)
Supplement: Supplementary file 1 [file cells-15-01944-s001.zip › cells-4571836-supplementary.pdf]

Endothelium-out (Geuder Glass Injector)

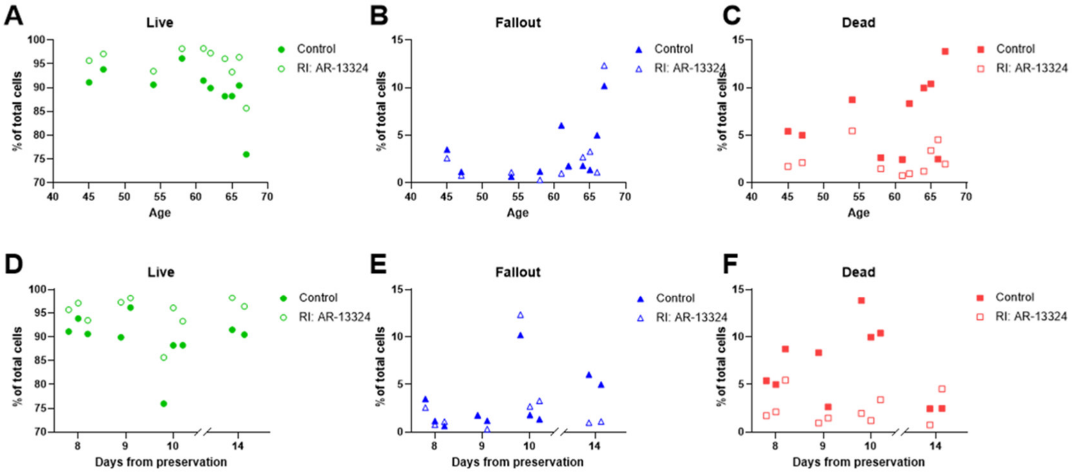

AGE	Live	Fallout	Dead
Control	Pearson's r = -0.5222<br>*p* = 0.1215	Pearson's r = 0.4088<br>*p* = 0.1215	Pearson's r = 0.3450<br>*p* = 0.3289
RI: AR-13324	Pearson's r = -0.3276<br>*p* = 0.3554	Pearson's r = 0.3966<br>*p* = 0.2565	Pearson's r = 0.02175<br>*p* = 0.9254

PRESERVATION	Live	Fallout	Dead
Control	Pearson's r = -0.06095<br>*p* = 0.8672	Pearson's r = 0.4546<br>*p* = 0.1868	Pearson's r = -0.3753<br>*p* = 0.2852
RI: AR-13324	Pearson's r = 0.1497<br>*p* = 0.6797	Pearson's r = -0.06669<br>*p* = 0.8548	Pearson's r = 0.06207<br>*p* = 0.8647

Supplementary Figure S1. AR-13324-treated tissues showed higher survivability and less fallout using the Endothelium-out technique (Geuder glass injector) regardless of age or days in preservation media. (A-C) Scatterplots depict percentage of live, fallout, and dead cells relative to total cell count following the procedure, against donor age. (D-F) Scatterplots depict live, fallout, and dead cell percentage against the number of days in preservation media before the day of procedure. Solid symbols indicate control tissues while outlined symbols represent treated tissues. Pearson correlation coefficients and respective p-values are given in the table above. N=10 matched paired donors were used. The tissues were stained with Calcein-AM and Ethidium homodimer-1 following the procedure and analyzed using ImageJ.

Endothelium-In (DMEK Endoglide)

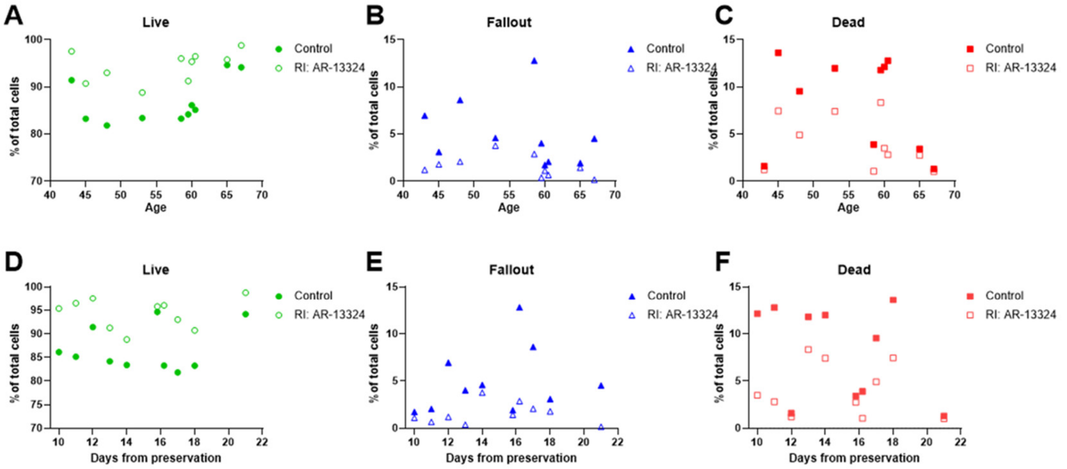

AGE	Live	Fallout	Dead
Control	Pearson's r = 0.4226<br>*p* = 0.2237	Pearson's r = -0.2862<br>*p* = 0.4228	Pearson's r = -0.2039<br>*p* = 0.5720
RI: AR-13324	Pearson's r = 0.3881<br>*p* = 0.2678	Pearson's r = -0.3787<br>*p* = 0.2805	Pearson's r = -0.2977<br>*p* = 0.4035

PRESERVATION	Live	Fallout	Dead
Control	Pearson's r = 0.2036<br>*p* = 0.5725	Pearson's r = 0.2794<br>*p* = 0.4343	Pearson's r = -0.3907<br>*p* = 0.2644
RI: AR-13324	Pearson's r = 0.04933<br>*p* = 0.8924	Pearson's r = 0.04740<br>*p* = 0.8965	Pearson's r = -0.07595<br>*p* = 0.8348

Supplementary Figure S2. AR-13324-treated tissues showed higher survivability and reduced fallout using the Endothelium-in technique (DMEK endoglide) regardless of age or days in preservation media. (A-C) Scatterplots depict percentage of live, fallout, and dead cells relative to total cell count following the procedure, against donor age. (D-F) Scatterplots depict live, fallout, and dead cell percentage against the number of days in preservation media before the day of procedure. Solid symbols indicate control tissues while outlined symbols represent treated tissues. Pearson correlation coefficients and respective p-values are given in the table above. N=10 matched paired donors were used. The tissues were stained with Calcein-AM and Ethidium Homodimer-1 following the procedure and analyzed using ImageJ.

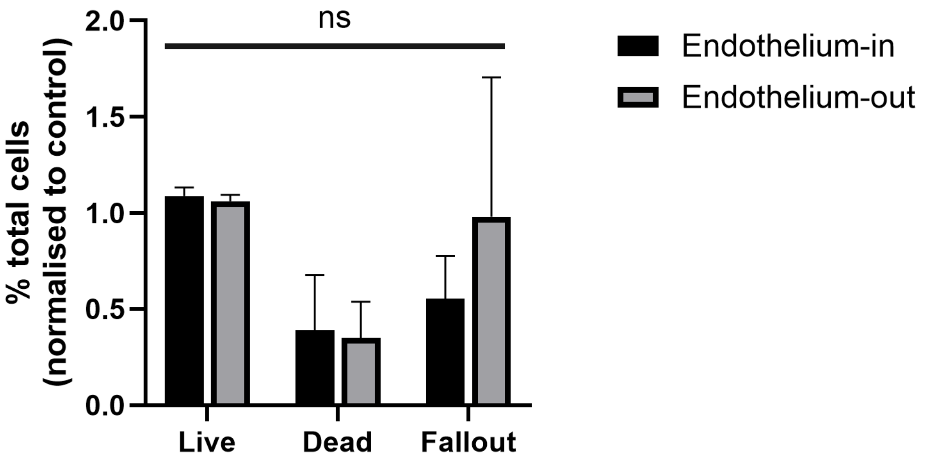

Supplementary Figure S3. Comparison of normalized values between the endothelium-in (Endo-in) and endothelium-out (Endo-out) methods for the AR-13324 treated group.

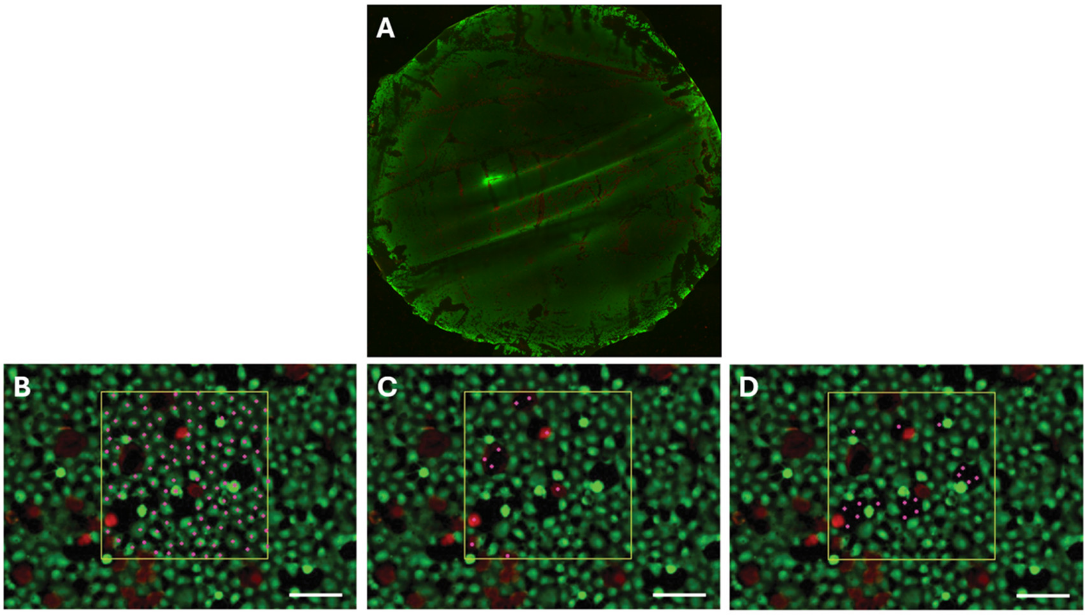

Supplementary Figure S4. Example of live/dead cell viability staining for corneal endothelial cell viability post-Descemet's stripping using Calcein-AM and Ethidium homodimer-1. (A) Stitched whole image of stained graft. (B-D) Inlays showing respective regions of interest with dots indicating (B) live cells (green fluorescence), (C) dead cells (red fluorescence), and (D) fallout (empty areas). Scale bar represents 100μm.
